# Remediation of at-risk medical students: theory in action

**DOI:** 10.1186/1472-6920-13-132

**Published:** 2013-09-27

**Authors:** Kalman A Winston, Cees PM Van Der Vleuten, Albert JJA Scherpbier

**Affiliations:** 1 Study Skills Centre, Room 202, Main Arts Library, College Road, Bangor University, Bangor, Gwynedd LL57 2DF, UK; 2 Chair, Department of Educational Development and Research, Maastricht University, Maastricht Netherlands; 3 Dean, Faculty of Health, Medicine and Life Sciences, Maastricht University, Maastricht Netherlands

**Keywords:** At-risk students, Remediation, Small group teaching, Classroom discourse, Facilitation skills, Teaching experience, Pedagogical context knowledge

## Abstract

**Background:**

Previous work has shown that a programme that draws on a blend of theories makes a positive difference to outcomes for students who fail and repeat their first semester at medical school. Exploration of student and teacher perspectives revealed that remediation of struggling medical students can be achieved through a cognitive apprenticeship within a small community of inquiry. This community needs expert teachers capable of performing a unique combination of roles (facilitator, nurturing mentor, disciplinarian, diagnostician and role model), with high levels of teaching presence and practical wisdom. Yet, despite participants’ convergent opinions on the elements of effective remediation, significant differences were found between outcomes of students working with experienced and inexperienced teachers. The current study explores the actual practice of teachers on this remediation course, aiming to exemplify elements of our theory of remediation and explore differences between teachers.

**Methods:**

Since it is in the classroom context that the interactions that constitute the complex process of remediation emerge, this practice-based research has focused on direct observation of classroom teaching. Nineteen hours of small group sessions were recorded and transcribed. Drawing on ethnography and sociocultural discourse analysis, selected samples of talk-in-context demonstrate how the various elements of remediation play out in practice, highlighting aspects that are most effective, and identifying differences between experienced and novice teachers.

**Results:**

Long-term student outcomes are strongly correlated to teacher experience (r, 0.81). Compared to inexperienced teachers, experienced teachers provide more challenging, disruptive facilitation, and take a dialogic stance that encourages more collaborative group dynamics. They are more expert at diagnosing cognitive errors, provide frequent metacognitive time-outs and make explicit links across the curriculum.

**Conclusions:**

Remediation is effective in small groups where dialogue is used for collaborative knowledge construction and social regulation. This requires facilitation by experienced teachers who attend to details of both content and process, and use timely interventions to foster curiosity and the will to learn. These teachers should actively challenge students’ language use, logical inconsistencies and uncertainties, problematize their assumptions, and provide a metacognitive regulatory voice that can generate attitudinal shifts and nurture the development of independent critical thinkers.

## Background

Throughout higher education, the current increase in both numbers and diversity of students brings with it concerns about maintaining retention rates [1]. When combined with commonly-voiced concerns that many students entering higher education are ill-equipped for the rigour of academic work, the need for active efforts to support these at-risk students is clear. However, there is considerable uncertainty about how best to provide this support, and about the precise roles of study skills centres and teachers in these efforts [2]. Within medical education, likewise experiencing an upsurge in enrolment [3], this has been accompanied by increased research into the efficacy of remediation for underperforming students, with a recent review noting the need for further development and examination of the details of complex interventions [4].

This link between widening access and high attrition is exemplified in our own context, a Caribbean-based US medical school which accepts students from diverse ethnic and socioeconomic backgrounds, most of whom have already failed to gain entry into mainland US medical schools. Our lower admissions criteria (mean MCAT (Medical College Admissions Test) of 23, compared to US mean of 31) contribute to an attrition rate of almost 30%. In attempting to address this challenge, we have developed an Essential Lifelong Learning Skills (ELLS) course, a programme drawing on a blend of constructivist, sociocultural and complexity theories that has resulted in significantly improved long-term outcomes for medical students who fail and repeat their first semester [5]. The ELLS course involves faculty-facilitated groups of six students meeting throughout a semester to work through a syllabus that integrates learning study skills and critical thinking with the content of basic science course material [5]. Exploration of student and teacher perspectives [6,7] enabled us to build a theory of successful remediation: participation needs to be mandated [6], and should provide cognitive and affective support within a small community of inquiry that motivates and challenges the students using a syllabus engendering self-regulatory, metacognitive, and dialogic techniques in small stable groups that meet a sufficient number of times to establish the trusting relationships necessary for the provision and acceptance of effective feedback and the development of more effective academic practices. This community needs a student-centred environment that fosters curiosity and joy for learning with teachers capable of performing a unique combination of roles: facilitator, mentor, disciplinarian, diagnostician and role model [7].

The opinions of all participants converged upon the aforementioned elements of remediation: students and teachers agreed on the ingredients required for success, and that all the teachers were performing these roles. And yet, while groups facilitated by all the programme’s teachers statistically outperformed historical controls, highly significant differences were found between those working with experienced and inexperienced teachers in retention and pass rates four semesters after taking this course [7]. This led to the conclusion that effective negotiation of the complex, context-dependent remediation process demands high levels of teaching presence and practical wisdom - the teaching and contextual expertise that comes with long experience. That teachers are key to educational success is not new [8,9], and neither is the finding that experience matters [10], since development of expertise, practical wisdom and teaching presence is a lifelong process [11-13]. The significance lies in the fact that it is typical to delegate remediation work to inexperienced teachers or student tutors, despite lack of improved long-term outcomes in such ventures [14,15] and evidence that experience may be required to maximize the effect of academic support [7]. However, when teachers themselves use similar language to interpret their own behaviours while their students attain differing outcomes, and when so many studies substitute self-report measures of satisfaction or belief for measures of performance, how are practitioners and administrators to distinguish who and what is most likely to be effective?

Many have questioned the relationship between teachers’ beliefs and their actions [16,17], noting the challenge of accurately articulating practice [18] and of enacting beliefs and principles [19-21]. Furthermore, researchers can only impose their own artificial categories on their findings [22,23], and, no matter how carefully the vocabulary they choose is explicitly operationalized [24], readers can only interpret from their own perspectives [25]. Thus it is no surprise that the education community has trouble pinning down and disseminating what counts as good teaching, and the kinds of questions we have received at workshops on underperforming students have served to underline the wide gap between what is described and what is understood.

Since education is complex [26], and small groups are complex systems [6,27], it is from interactions that characteristics emerge [28-30]. And so, having generated ideas about a theory of remediation [5-7], for clarification of exactly how it works [31] we must look carefully at classroom life to verify our findings [25,32] by exploring actual behaviour in practice [17,21,33,34].

### Aims

The current study examines events in the classrooms of the remediation course for at-risk medical students previously described by Winston et al. [5]. In doing so, we have two intertwined aims. The first is to use the interplay between our theory of remediation and our practice [35-37] both to verify and test what we have described [25], and to illuminate and extend our understanding of how remediation works. By illustrating and exploring more deeply the context of our efforts [38] and documenting examples of our practice [18], we hope that others can come to fully appreciate this work, and then test and extend the ideas in their own contexts. Thus, our first research question is:

*To what extent can examples of remediation practice be used to illuminate and extend the theory of remediation*?

The second aim is to seek out differences between experienced and inexperienced teachers’ classroom interactions. If differences are identified, they may help explain the noted variation in outcomes [7], and provide valuable tools for training novice teachers and promoting development of their practical wisdom [39]. Indeed, this should feed into the first aim by informing our theoretical knowledge: as Shulman [18] so eloquently stated, “The neophyte’s stumble becomes the scholar’s window.” So, our second research question is:

*What are the differences between experienced and inexperienced teachers of remediation, and how can these differences deepen our understanding of effective remediation*?

Answers to this second research question will, by extension, help answer the first, by providing clear examples of variation in remediation practice.

## Methods

Given these aims, this research is necessarily mostly qualitative, as we attempt to provide a rich contextual description that elaborates details of our intervention [40,41]. Still, we have also continued to track quantitative performance data reported previously [5,7], specifically the students’ basic science completion rates, four semesters after participation in our Essential Lifelong Learning Skills (ELLS) course for students repeating a semester at our medical school.

The lead author, KW, observed and audio-recorded twelve small group sessions led by six different teachers (including KW) working with 49 different students during their participation in ELLS. Eight separate groups of five to seven students were studied (four groups were observed twice), and each small group session lasted approximately 90 minutes. The work was ethically approved by Ross University’s Institutional Review Board, and all participants gave informed consent. The resultant nineteen hours of recordings were then transcribed, with repeated careful listening revealing ever more detail [9], to yield 564 pages of transcript.

Audio-recordings were preferred to video recordings for three main reasons: firstly, participants were more comfortable with less intrusive audio-recording [42]; secondly, one small digital sound recorder enables inclusive capture of all participants’ speech, whereas multiple cameras and angles are required to include everyone via video; and, while gesture, gaze and facial expression are undoubtedly important elements of group dynamics, focusing on dialogue allowed us to keep the scope of the project manageable. Indeed, dialogue is the essence of education: it is through dialogic interaction that knowledge emerges [35,43], through discourse that we think and create meaning [44,45]. There is evidence that the way talk is used in classrooms affects learning [46], and analysis of classroom talk is core to understanding the effects of remedial interventions [47].

Of course, one’s choice of method for talk analysis is not neutral, and inevitably affects one’s findings [48]. Since our aim is to disambiguate, rather than proliferate, the categories that are evidently open to different interpretations, we have chosen not to create and enumerate codes that divorce the content of talk from its functional context [46,49]. Instead, we treat discourse as a situated, action-oriented tool for the construction of interaction and shared understanding [50,51]. Just as seeing a tool in use enables clearer understanding than merely hearing a description, we provide representative samples of classroom talk in context as integral to comprehending the application of remediation theory in practice: the medium is very much part of the message. We take a pragmatic approach, drawing on sociocultural discourse analysis [46], in which the content and function of language are analysed and interpreted, in order to understand how participants’ learning and behaviours shape and are shaped by classroom interactions [46,52].

With such a large amount of data, selections have to be made. Through multiple readings of the transcripts, talk samples have been chosen to illustrate some of the thematic categories previously described in our theory of remediation, samples that seem most suitable for crystallizing understanding. We have also identified talk samples that exemplify newly emergent ideas about differences between experienced and inexperienced teachers [25,53-55]. In our results, the talk samples are numbered as TS1, TS2, etc.; turns at talk, which, as the basis of dialogue [56,57], we take as our fundamental unit of analysis, are numbered as Utterances (U). Students within each group are labelled as S1, S2, etc., while teachers are labelled according to Table 1.

**Table 1:** Teachers’ experience and their students’ long term outcomes

**Teacher**	**Highest qualification**	**Teaching experience (yrs)**	**Context experience (yrs)**	**% students passing 4th semester**
T1	MSc	4	2	35% (n = 20)
T2	PhD	5	1	36% (11)
T3	MD	5	3	44% (66)
T4	MBBS	15	3	50% (14)
T5	MEd	28	10	70% (162)
T6	MEd,MA	40+	7	62% (139)
T7*	DVM	3	3	54% (90)
T8*	PhD	4	2	51% (72)
T9*	PhD	39	12	60% (25)

*left the school prior to this study, but was included in the previous study, Winston et al., [7].

In such an analysis, the researcher, as interpreter of discourse, is very much part of the method [58], and objectivity is clearly a challenge when the principal investigator (KW) is also one of the subjects. However, ‘objectivity is a chimera’ [59]: all observers bring their own paradigms to their perceptions [25,60], and outsider researchers’ unfamiliarity with the context of their subjects often leads to misunderstandings [42,61] that can ultimately serve to distance researchers from practitioners. Hence the many calls for practitioner research, which affords the opportunity for insider exploration of one case study in enough detail for others to draw lessons applicable to their own contexts [38], and may thus foster generalization and integration of theory and practice [62,63]. So, while the analysis admittedly follows an interpretivist approach suffused with a practitioner-researcher’s thoughts, opinions and experience [64] in one specific context, hopefully some clarity emerges for the broader practice of supporting at-risk students in their academic work.

## Results

Table 1 presents some data pertaining to the teachers involved in the study. The final column shows the percentage of students who, having worked with a particular teacher during their repeat of first semester, went on to successfully complete the basic science curriculum at the end of their fourth semester. The correlation between context experience, operationalized as number of years teaching at this school, and long-term pass rate is 0.81. The correlation between total teaching experience and long-term pass rate is 0.72. These are high, suggesting that 51% – 65% of the variance in student outcome could be due to teacher experience. While many other factors must be involved, this is worth exploring.

A number of sharp differences between teachers emerged from the recordings and observations. For example, one of the students’ favourite exercises [6] is working with practice multiple-choice questions (MCQ). After reading the question stem, the experienced teachers (T5 and T6) insist that their groups rephrase the question and discuss it in detail, typically spending around ten minutes exploring definitions and relations between terms, thoroughly eliciting ideas before allowing their groups to consider the answer choices. The other teachers averaged about one minute doing this. This difference is reminiscent of the way experts slow down and take more time planning their approach to problems than novices [65,66]. Talk Sample 1 (TS1) demonstrates how this can play out in the classroom. Note the lengthy silence at Utterance 3 (TS1.U3): expecting answers to questions, and waiting for them, is a key element of rigorous facilitation. At TS1.U6 the teacher observes the difference between the two responses, a move that required attentive listening from the teacher, and exposes a clear misunderstanding. On the rare occasions that inexperienced teachers required rephrasing of the question, they did not go on to press the students to explore differences between their versions. In U8 we see a diagnostic intervention, a challenge for specificity, a query that is reiterated in U16: this tenacity, the refusal to let a question go unanswered, seems to be a hallmark of the expert teachers rarely displayed by their less experienced counterparts. In U12 we see a deliberate attempt to draw another group member into the conversation, and likewise in U26, where other group members are asked to verify the accuracy of S2’s contribution: noteworthy here is that the facilitator neither provides the answers, nor acts as the verifier of content accuracy. This typically results in the group resolving their own understanding, self-correcting (U11, U14) and supporting each other’s ideas (U13, U27), especially when invited to predict the answer (U24). The sequence at U19 to U22 shows the student asked to finally reformulate the question indulging in light-hearted mimicry, playfulness that is encouraged by the teacher and aids group cohesion, a type of behaviour that actually occurred in the groups of all the teachers: the nurturing mentor role seems much easier for the inexperienced teachers than the roles of disruptive facilitator, disciplinarian and diagnostician.

Talk Sample 1. T5, Working with a multiple choice question (MCQ), 7th group meeting:

1.) T5: Ok, so read the question for us.

2.) S1: Okay, so Phosphoinositide-3-kinase forms inositol phospholipids that are phosphorylated in position 3. Normally, this phosphate group is removed by the lipid phosphatase PTEN. When a cell loses PTEN through somatic mutations, the most likely effect of this will be?

3.) T5: Okay, so let’s rephrase the question. [*20 seconds silence*]

4.) S2: Okay, when a cell cannot remove a phosphate group, this would most likely lead to?

5.) S3: When the cell can’t remove the phosphoinos 3 kinase that will result into?

6.) T5: Okay, those are very different.

7.) S3: Yes. I just figured if you can’t remove the phosphate you can’t get rid of the phosphoinositol-3-kinase.

8.) T: What is it you can’t remove the phosphate from?

9.) S3: From PI3 kinase, you have to remove it, it has a phosphate on the third position, right?

10.) T: Is there a problem reading it that way?

11.) S3: No, it’s not that, I’m sorry. It forms phospholipids that are phosphorylated on the third position, because of the kinase. I’m sorry.

12.) T5: So how would you rephrase this? [*turning to s4; another long silence ensues*]

13.) S4: I would do the same way she did, if you can’t remove the phosphate group, what happens?

14.) S2: But I made a mistake too, because it has to be IP3 pathway, because if it’s anywhere else, it’s different.

15.) S3: So it’s a different pathway.

16.) T5: So if you don’t remove the phosphate group from what, I think that’s the piece that we’re missing.

17.) S3: It’s from the IP3?

18.) S4: Same thing as she said, just remember it’s the IP3 pathway.

19.) T5: So say it again then, but include that piece.

20.) S4: IP3 comma [*others laughing*]

21.) T5: Absolutely, go for it, yes.

22.) S4: IP3, if you, what was it, um, if you can’t remove the phosphate, what happens?

23.) S2: Yes, that’s right.

24.) T5: So then before reading the choices, what do you think would happen, what do you know about this pathway?

25.) S2: First I know, no matter what it is, it’s going to be an active pathway because sometimes when you phosphorylate things, it inactivates it, but this cascades, definitely something that will occur, something will be like stimulated, not inhibited.

26.) T5: Yes, no?

27.) S4: Yes, it’s just the two things that get, the IP3 phosphorylates them, the PKB or the AKT, just keeps getting phosphorylated, so it constantly kicks them onto the cascade, like nonstop.

28.) T5: Okay. So now let’s go through the choices one at a time.

Talk Sample 2. T5, After answering the MCQ from TS1, later in 7th group meeting:

1.) T5: But again, it’s important to be clear what the question is getting at, because it was very densely worded, wasn’t it?

2.) S4: Yeah, it was, like I have no idea what PT and EN means.

3.) S2, S5: Me too

4.) T5: But you didn’t need to know, because it told you what it does.

5.) S6: That’s when I like, because I have it all the time, like I see a word that I’ve never seen before and I freak out.

6.) T5: This is why this exercise of rephrasing is so important. In the previous question we were talking about with seizures, you haven’t done seizures really yet, but it told you what seizures are in the question. Here, he’s told you what PTEN does, so you went straight to the point and said, okay, we’re not removing the phosphate, that was good.

TS2 is a sequence occurring after discussion of the answer choices has led to resolution of the question. Before allowing the group to move on, there is a recap of what happened. In U1, the challenge they faced at the beginning is noted, with the ‘dense wording’ offered as an explanation. This ploy allows group members the space to admit difficulties, a prerequisite for improvement: we see three students voice agreement about the problem (U2, U3), and this encourages S6 to elaborate on his anxiety (U5). The teacher then summarizes the importance of the skill and reminds them of a similar example from which they can begin to generalise, while also finding a way to praise, very specifically, what they did well. These ‘metacognitive time-outs’ for attending to process happened multiple times in the classrooms of experienced teachers, but almost never with the inexperienced teachers.

TS3 and TS4 offer a contrast to the previous two samples. As before, a student has just read a multiple choice question written by that student as a homework assignment. TS3.U1 is a good opening move, inviting discussion of the question format, as is the follow-up question (U4). However, accepting that it is clear to everyone on the response of one student (U5) is a missed opportunity to uncover any misunderstandings in the group: she could then have asked others to rephrase the question stem, or probed them to predict the answer before examining the answer choices, moves that elicit careful thought and expose uncertainty. Likewise, the initial moves in TS4 are positive (U1, U3), eliciting student reflection on the construction of the question (U4). The teacher then nurturingly validates the student’s framing of her approach to learning – the unproductive classroom game of guessing what the lecturer wants [67], going as far as to suggest that this is their goal (U7). Yet this should most definitely not be their long-term goal. The real challenge is to inculcate a curiosity, an approach to learning that will carry them throughout their careers. Indeed, even if the object were to ‘get inside the lecturer’s head’, the best way to guess how an expert thinks is surely to learn to think as an expert. In this example, there was an excellent opportunity to press this point. In TS3, the students have agreed that this is a straight recall question, but in TS4.U4 the student claims her aim was to create a secondary question. A really helpful move would have been to call them on this, and then get the group to re-create the question together, to discuss what a secondary question actually looks like, and implement the wording collaboratively. In this particular example they could easily have replaced the answer choices stating anatomical structures for choices describing clinical deficits ensuing from damage to those structures. Indeed, T6, when dissatisfied with the questions produced by her group, had the students step back, identify the organisational structure and key points of the relevant lecture, and then produce a question together on the whiteboard. Seeing this opportunity in the moment of classroom action partly depends on a kind of contextual expertise, on having seen discussion of enough questions to know the type students struggle with, and partly on the adaptability and presence to follow through on those teaching moments that arise spontaneously as students expose their knowledge and shortcomings.

Talk Sample 3.) *T2, Working with an MCQ, 7th group meeting:*

1. T2: How does that question sound before you go on answering it?

2. S1: It’s a memorization question.

3. S3: Recall.

4. T2: Is it clear though?

5. S3: Yes, it’s pretty clear.

6. T2: Then looking at the choices, what about a)?

Talk Sample 4.) *T2, After answering the MCQ from TS3, later in 7th group meeting:*

1. T2: And S1, how was it going through the man being stabbed?

2. S1: I tried to hit three different topics so I did anatomy, then did one embryo, and then one on neural tissue so I was just trying to spread it out evenly.

3. T2: But then how you actually wrote it though, taking that …?

4. S1: I tried to make it similar to a secondary question they would ask on a mini because I know Dr.X loves those questions about a man was shot here, stabbed here or fell on this portion of his arm, what would be the deficit seen?

5. T2: So you got into her head a little bit with how she props up the questions…

6. S1: Tried to, yeah.

7. T2: That’s our goal as a practice, right? All right.

Talk Sample 5.) *T6, 22nd group meeting*

1. S1: I’ll tell you what, Y’s lecture was heavy yesterday. Really heavy.

2. S5: I like her lecture though.

3. S1: I don’t like developmental for some reason. Embryology is like, so we came about, we’re here, now what. I mean we’ve got to learn it.

4. T6: Does anyone love the embryology?

5. S4: I do.

6. T6: What do you see in it, that she’s hating.

7. S4: I don’t know. I mean it’s just, a lot of diseases that we can’t cure yet are congenital, embryological

8. S1: Yeah

9. S4: And if you can’t repair it surgically, then there’s really no cure for it. Or it’s an enzyme defect, so, I don’t know. It’s like a frontier, it’s like neurology, people don’t really know everything about it.

10. S1: I think it’s because we can’t do as much about it too, you know what I mean?

11. T6: It’s also very hard to visualize

12. S1: It is, it’s very hard to visualize

13. S2: Embryology? Yes. I’ve been looking for embryology videos since first semester.

TS5 is an example of how one can help foster curiosity, the joy of learning for its own sake, so absent in TS4. After the comment about disliking embryology (U3), there was a danger of the group focusing on unhelpful negativity, but this is expertly refocused towards appreciative enquiry (U4), especially when S4 is encouraged to elaborate on his love of embryology (U6). Engaging the group in this kind of positive social regulation [68] is invaluable for students struggling with the stigma of failure, especially when displays of intellectual enthusiasm are so often disparaged among modern students [51]. At the same time the teacher manages to validate the challenge they face (U11), which draws in another student to the conversation (U13). This last comment provides a space for sharing of resources – it just so happens that this teacher has been collecting links to embryology videos for several years, another facet of her contextual experience.

TS6, on the other hand, shows one student interacting with the teacher in a conversation about their time-management assignment. This sample exemplifies the initiation-response-feedback (IRF) format of classroom dialogue that positions the teacher as the authority in the classroom [69]. This was the dominant form of discourse in the groups of less experienced teachers, with students taking turns to engage with the teacher. Although there was some student-student interaction in discussions of content, conversations about process (with T1 – T4) were almost exclusively in this IRF format that does so little to promote the social-regulatory group dynamic of a genuine enquiry-based classroom. In TS6.U3 the teacher does a good job of checking whether the student attempted to stick to the schedule he made, but then, after hearing the student’s explanation (U4), the suggestion is to simply try more of the same (U5), which gets a predictable non-committal response (U6). Instead, T4 could have picked up on the fact that the student has twice said his schedule is too dense (U2, U4), and asked him to point out a specific example. If they had then focused on this, and other group members had been invited to offer potential solutions, everyone would have been more engaged. The comment at U8, however, implies that perhaps his schedule was not so dense after all, and indeed the student appears somewhat defensive, positioning himself in opposition to ‘you guys’. Although the teacher then does well to validate his stance and behaviours (U9), he could, for example, have gone on to highlight one section of his schedule, and then asked the group to help re-work it towards something more realistic for him. And then, when the student reveals he has produced a new version for the coming week (U10), the teacher doesn’t ask him to share or compare this new version with the one they have discussed, but simply moves on to the next student, thus missing the opportunity to further practice the self-regulatory processes of reflection and planning.

Talk Sample 6.) *T4, time management, 3rd group meeting*

1.) T4: And how about yours?

2.) S1: I’m not good with the schedule thing. I mean, I wrote stuff out, but it’s too dense and there’s no way I can stick to the schedule that I made.

3.) T4: You tried that?

4.) S1: Yeah, I tried, I mean I tried, it’s just impossible cos I think I make it too dense. And I want to do all this stuff and it’s like, well I’m gonna add another hour to that, and I’m gonna add another hour to that, and this and that, but I’m better off listing what I’m going to get done during the day and having my blocks of study.

5.) T4: I think, you have only made two weeks that you have completed this way, so you can give a try again and see how it goes.

6.) S1: Ok.

7.) T4: So in that whole one week you could not achieve what you wanted in that week?

8.) S1: Well I mean I study, I touch on everything, but it’s just like a matter of, you know, how successful you can be. That’s why I do it in two hour blocks. You guys say to do it in one hour blocks, but I give myself 2 hours because I know in that 2 hours I might take a break or I might, not like take a break as in go off to eat, but take a break mentally, or you know, just kind of add in some leeway here.

9.) T4: That’s ok. You need those things, I don’t disagree at all. His schedule is even more detailed, he has filled it with minute things every hour. Really great if you can follow this actually.

10.) S1: I think it would be, that’s why I tried. Last night I did the one for this week.

Talk Sample 7.) *T5, drawing, 21st group meeting*

1. S2: That was the point I was going to bring up after you answered this question because all, when these, the development of all these vessels, it’s like it’s just pretty much memorisation. I was just wondering if you guys have any easier way to memorise all that or not memorise but remember all that.

2. S6: I just like drew it out.

3. S2: Yes, I drew it out last semester, but it didn’t help.

4. T5: Let’s see it.

5. S2: I didn’t actually draw it out. I just wrote it out.

6. T5: You drew it out? [*Directed at S6*]

7. S6: Yes, like this one’s really easy. You either know it or you don’t. Like, her pictures are really small, but they make a lot of sense. [*And he gets up, starts drawing on board, and explaining as he goes (cardinal veins, etc.), with others chipping in to add to his explanation. It takes less than a minute.*] …

8. S5: Yeah, you just have to figure out if it’s left or right, and then you can remember pictures.

9. S2: Yeah, that would have helped me, cos it just broke it down.

10. T5: That picture was good.

11. S2: I guess I can try that. But this is going to take time for me.

12. T5: How long did it take him to draw that? *[ss laughing]*

13. S2: I don’t know. I just… Because I would have to sit down for all these other things, draw it out.

14. T5: Do you remember that conversation we had a couple of weeks ago?

15. S6: Well, like, this is what I’m saying, if you draw it out, the SSVC, then this one makes sense. I had to draw it out, otherwise I won’t remember it.

16. S5: It only took me thirty minutes. I maximised the screen as much as I could, and then I drew it out. The pictures are really good.

17. S2: Yeah.

18. S5: Like the same thing with the superior mediastinum. There’s a lot of information. It took me 20 minutes to do this thing because I can’t draw but I have to like draw it properly. It took me only 15 minutes to draw and after that I just knew where it is. Because you draw it based on what it says, like in words, but I can’t memorize the words.

19. T5: It’s twice in a session we’ve had a drawing suddenly make it clear. That was really quick and clear. And then, when you drew your arrows, that was the moment when everyone goes “oh yeah”. *[laughter, general agreement]* It doesn’t take long to do it, and it doesn’t have to be beautiful.

20. S2: Yeah, no, I know.

Some effects of promoting group discussion and reflection on process are illustrated in TS7. We join the group after a question on embryological development of blood vessels has been resolved. At this late stage of the semester, students have become comfortable raising questions about both content and process in the group, as we see in TS7.U1. After S6 responds with his method of drawing (U2), we see U3 claim that drawing didn’t help. At U4, the teacher asks to see his earlier work, a suggestion with a powerful purpose: struggling students frequently claim that a method didn’t work for them, typically because of either ineffective or non-existent implementation. In the former case, displaying his drawing would have enabled the group to examine it and identify ways to improve it. In this case, however, we see the latter outcome, affording the opportunity to enlist a group effort (U6). For the remainder of this segment, we see the efforts that S2’s colleagues make to persuade him of the value of drawing, spontaneously creating a drawing on the board, and reinforcing each other’s explanation. Note, for example, how S5 (in U16) picks up on S6’s earlier comment about the small size of the image in the lecture (U7), provides a solution, and even praises the quality of the material. This last point is a real achievement: getting students who failed to appreciate the positives in lecture material, and find workarounds for the less effective parts, is an important attitudinal shift from the common starting point of blaming the poor quality of the teaching for their struggles. After the teacher has praised these efforts (U10), S2 is still hedging (U11). This is common – a student agreeing a method is a good idea, but then quickly rationalising why perhaps *he* still might not do it. It’s important to call them on this, as T5 does in U12, a move lent support by the subsequent contributions of S5 and S6. In response to further resistance (U13), the gentle reminder of an earlier discussion (U14) about the importance of fully exploring ideas and adapting them to their own needs is a pointer to the importance of group continuity and stability, and is part of the disciplinary and mentorial aspects of the teacher’s role. The sample ends with another example of the metacognitive generalisation by experienced teachers mentioned earlier, with T5 highlighting how effective S2’s earlier drawing to explain blood flow through the heart had been in creating group understanding. Indeed, this particular student, s2, was, eventually, so thoroughly convinced that when he reached his fourth semester he volunteered to speak to the new batch of repeating first semesters about the importance of fully participating in the course.

Talk samples 8 and 9 highlight the kinds of confusion that can develop when students are pressed to explore content in detail. In TS8, the group has settled on an answer to a biochemistry question they have been discussing. The teacher’s response (U2) to the opening question (U1) effectively problematizes an issue the students had earlier sidestepped – they had been unable to find a clear reason to eliminate one of the wrong answer choices, ferrochelatase. However, instead of affirming the correct answer with ‘yeah’, she could have invited the whole group to consider their confidence in their chosen answer choice, and to identify for themselves what they were unsure of: earlier in the course the students are introduced to confidence marking [70,71] and encouraging regular practice of this calibration technique aids development of students’ self-regulatory habits. Nevertheless, T1 has successfully exposed a major misunderstanding of the structure of hemoglobin that goes way beyond the function of this particular enzyme (ferrochelatase). Allowing this confusion to play out between the students is certainly reasonable, but sometimes facilitator intervention is helpful. For example, when S4 disagrees (U9) with the preceding statement, it may have been wise to flag this disagreement and ask them to make their positions explicit. More troubling is the failure to challenge the inconsistency of S4’s statements at U6 and U12, both of which receive support from other group members. The logic of the two statements taken together could imply that protoporphyrin and iron are the same thing, and yet the group would certainly not agree with that proposition. Drawing the group’s attention to this would have caused them to rethink their argument much more carefully; doing this repeatedly through a whole semester can go a long way towards helping them to hone their critical thinking skills. The ability to catch these errors often appears, to observers, to be about the teacher’s content knowledge, but in fact it depends more on attentiveness, curiosity, and the ability to follow the logic of students’ argument; in this study, the two most experienced teachers (T5 and T6) have ‘lower’ content-related qualifications than their colleagues.

Talk Sample 8.) *T1, 10th group meeting*

1. S2: Is C the right answer then?

2. T1: Yeah, but back to ferrochelatase. Tell me what it does.

3. S1: So you have like your iron molecule, and like your heme molecule, which is also called protoporphyrin 9.

4. S3: Yeah

5. S1: And then that combines the two together. That’s like the iron to the heme, and it makes, like, hemoglobin. No, not hemoglobin. Is it?

6. S4: You’re adding heme to protoporphyrin, so it is hemoglobin.

7. S2: Yeah, so it is hemoglobin.

8. S1: No, heme is the same thing as protoporphyrin.

9. S4: No.

10. S1: Iron is put into heme. Heme and iron make up

11. S3: Heme and iron make up hemoglobin.

12. S4: Yes. So four subunits of heme plus an iron make up hemoglobin.

13. S5: Yeah.

14. S4: Okay, so this guy, ferrochelatase, makes, you said, heme plus

15. S1: I’m pretty sure, it’s like the last step, right? Do you know?

16. S3: I don’t remember.

17. S1: It’s either that one or the one before. I’ll check. I just looked at it too.

18. T1: You just looked at it?

19. S1: Yeah. And now I’m second guessing myself.

20. T1: Well, when you review, then you’ll never forget.

TS8 ends with another major missed opportunity, as T1 optimistically hopes that a later review (which may or may not happen) will suffice (U20), instead of unpacking what has just happened and pursuing an alternative approach to understanding. Early in the course there are sessions in which the explicit focus is on language and medical terminology, with assignments on definitions, analogy, and word roots. As with the confidence marking, T1 fails to link back to these longitudinal course themes. In this sequence, she could have directed the students to break down these terms into their roots (proto, heme, globin, chelate, etc.), a technique that most likely would have been enlightening. In the classrooms of experienced teachers, language becomes a major thread throughout the course, and they continuously raise students’ awareness of loose jargon, ambiguous pronouns, confused direction of causation, and unnoticed common word roots, thus enhancing both their content and process understanding.

TS9 illustrates this difference between teachers. The group has just read through a handout that introduces the importance of asking questions, and takes them through an exercise in which they ask each other questions about one lecture of their choice, using primary, secondary and tertiary questions. One of the students has asked how Parkinson’s disease might appear in a 65-year-old patient, and, as in TS8, the group have talked themselves into a thorough groupthink misunderstanding by U13. At U14, the teacher asks a really simple question, which is pressed at U16 and U20 and further simplified at U23, with justification for this probing given at U18. Notice how this elicits the admission that they don’t know the meaning of this term (U17, U21): everyday English can be as problematic as technical jargon. Then, when S2 actually draws and demonstrates, we get an in-class eureka moment (U25 – U29). This took less than a minute (from U14 to U25), and is clearly more effective than the way it was left hanging at the end of TS8.

Talk Sample 9.) *T5, asking questions, 3rd group meeting*

1. S5: I think there’s the gait

2. S1: cog-wheel

3. S2: It’s called cog-wheel rigidity.

4. S3: The gait is the shuffling gait.

5. S1: and there’s the pill-rolling. What’s the name for pill-rolling?

6. S5: It’s called pill-rolling

7. S1: That’s the cog-wheel?

8. S2: No, no, the cog wheel is like…

9. S1: so what is the pill–rolling?

10. S5: that is after. The first one is the patient won’t even know it, but the doctor will pick up on it when they come in, like he’ll notice a lack of…

11. S2: the cog-wheel rigidity, right?

12. S4: Yeah, that’s what cog-wheel rigidity is, the doctor notices but the patient is unaware.

13. S1: good deal.

14. T5: What is a cog-wheel?

15. S5: rigidity

16. T5: What is a cog-wheel?

17. S5: I don’t know exactly what a cog-wheel is.

18. T5: You keep skipping the fundamental questions, but I think they’re the most important.

19. S1: It’s the, er, I want to say it’s the reduced range of motion in the arm. That’s the cog-wheel rigidity.

20. T5: What is a cog-wheel? Or a cog?

21. S1: I have no idea.

22. S2: A cog is in a machine

23. T5: Exactly what is it?

24. S2: It’s er, you know the big old clocks? And then they have little rivets [*draws a cog wheel on the board]*. You know, and then they move, like this [*demonstrates jerky movement]*

25. Ss: oh [*sudden understanding all around]*

26. S4: That worked!

27. S3: Now I’ll never forget it.

28. S1: So it’s gonna move like a clock.

29. S5: Like a clock.

The remaining three talk samples, all taken from a single session in which the group is collaboratively creating a concept map of a challenging biochemistry lecture, draw out a number of the preceding themes. As preparation, students were asked to prepare a key word list for the lecture, but none had actually done so. The teacher responds adaptively, suggesting an alternative approach by inviting them to summarize the lecture, while noting, throughout the ensuing process, the points where the original approach would have been beneficial, as exemplified in TS10.U1. In this initial phase (TS10), it is often necessary to be somewhat directive, as at U9, and to ensure clarity, as with the reformulation offered in U12. Indeed, there were a couple of times during the session when the teacher went to the whiteboard to demonstrate how a just-discussed link or idea might be represented, revealing the possibilities of the technique. This level of interference is valuable in early stages of concept map training, as long as it is used sparingly, and is simply another example of facilitative reformulation and modelling. It is, of course, important to draw everyone in to the process, making U6 a key comment. The sequence from U13 to U19 is especially helpful with respect to this development of group ownership of the task. S2 has raised an objection (U13) to what has just gone on to the board, which despite apparent acknowledgement from S1 (U14), is ignored by S5 and is not being reflected in the drawing. The teacher’s reformulation at U17 works to establish ground rules and promote teamwork skills, validating the contribution from a quieter voice, and giving her the confidence and space to restate her point with clarity in U19. This noticing, of a quiet voice, tentative disagreement, or even just a puzzled facial expression, and then drawing that student to voice, or re-voice, her concern, is essential to building trust and a group dynamic where all contributions are valued, and willingly offered.

Talk Sample 10.) *T5, concept mapping initiation, 6th group meeting*

1. T5: So, if you’re gonna… this is the piece, for me the most important piece is to get an overall organisation, a structure to it. So, I’ve always found it easiest to see all the words in one space, but if you don’t want to do that, is it all one? Are there sections you can divide it into?

2. S4: The different types of receptors, and the different types of signalling cascades.

3. T5: Ok. That’s it? That’s the whole thing?

4. S4: That’s how I would divide it, is into the specific types

5. S6: And the pathways of the signals. Like divide into the specific types, then under each types you write the pathway that type takes in order to have an effect.

6. T5: Does everyone agree with that structure?

7. S3: Yeah.

8. S6: Cos that’s what he talked about, receptors, and, you know, how they respond to hormones. Like you have the receptor that is responding to hormone, but it just don’t respond, it has to go through a pathway, so he spent most of his time talking about those signalling pathways that it goes through, and what can happen. Like if this goes through three different steps, maybe if you stop it on this step, what’s going to happen.

9. T5: Ok. So if you were going to present this as a concept map to me, to show me this broad outline of the lecture, how would it look? Could we put that on the board?

10. S1: He divides them first, between the soluble receptors and the insoluble receptors, those that can come into the cell and those that cannot

11. S5: right

12. T5: So there’s two broad classes then. Ok, so write ‘receptor’ right in the middle there.

13. S2: Wouldn’t it be hormones that are soluble or insoluble. The receptors are, like, membrane bound or not

14. S1: Yeah. So soluble hormones would get into the cell

15. S2: right but

16. S5: but like location of receptors you could put

17. T5: But what S2 is saying is that it’s not receptors that are soluble, it’s the hormones

18. Ss: It’s the hormones.

19. S2: So I just mean that instead of having ‘receptors’ up there, shouldn’t that be ‘hormones’?

The group then gets involved in constructing their concept map, classifying, structuring, and thinking carefully about similarities and differences between pathways. Talk Sample 11 occurs about an hour into the process, when discussion has turned to the effects of cholera toxin. By this time, we see the group largely self-directing their work, providing their own suggestions about how best to represent their ideas (U2, U6), and prompts towards deeper exploration (U7) that are actively taken up (U8). Still, the teacher continues to ensure the students take care about the accuracy of their work (U4): pointing out the need for self-disciplined thinking, whether that is through verbal or physical representation, remains an essential element of rigorous facilitation. This demand for high expectations is exemplified at U10 and U12. Although the group has already agreed on how cholera toxin exerts its effect, the teacher inserts two disruptive questions to challenge their comfortable consensus. This again highlights the value of attentive listening, contextual expertise and group stability: in the previous week, this group had briefly mentioned cholera when discussing a question from a histology lecture. Here, after the problematization of their discussion (U12), we see the group in genuine collaborative knowledge construction, completing each other’s sentences (U13 – U24) as they work through the details of the process, culminating in S1 explicitly making the link between the two lectures (U22, U25). This realisation was followed by discussion about differences in level of detail between the two lectures, and proposals for bridging concepts across the two disciplines. Getting ‘weak’ students to make these kinds of links is real achievement, and is not uncommon in the groups of experienced teachers. In another example, a group was struggling with the concepts of preload and afterload in cardiac physiology: they were trying to solve a question about the left atrium, and were convinced the terms could only be applied to the ventricles, in relation to which the terms had been taught. A simple question from the teacher, “So there’s no pressure and volume in the atrium?” prompted lengthy discussion resulting in “I mean we learned about preload, afterload and contractility with just muscles. Yes, I think it can be applied to atrium as well.” For students who have been struggling, these carefully timed prompts have the power to transform their thinking and enhance the likelihood of knowledge transfer occurring.

Talk Sample 11.) *T5, concept mapping, 6th group meeting*

1. T5: Ok. So how are you going to capture that?

2. S2: You could write, like, cholera, and then write like a whole bunch of increase arrows to G_s_

3. S5: Ok, so [*writing on whiteboard]*

4. T5: To G_s_, not to just G. where you point it matters, doesn’t it? You can’t allow yourself to make that slight inaccuracy.

5. S5: Oh….cholera

6. S2: and just write some up arrows, there

7. S1: Do you want to write how exactly it has an effect? Cos he mentions that.

8. S5: ..to G_s_, and it causes high cAMP, which makes sense, and then high cAMP, should I say equals diarrhoea? Or high cAMP is watery diarrhoea?

9. S2, S3: Yeah

10. T5: Well, why does it give you watery diarrhoea?

11. S5: Cos you have too much cAMP.

12. T5: cAMP is watery?

13. S4: No, it erm

14. S1: it’s the way it acts on the intestine.

15. S3, S2: yeah

16. S1: Too much cAMP on the tissue itself

17. S5: causes

18. S1, S3: diarrhoea

19. S2: It’s cos er electrolyte

20. S5: imbalance.

21. S2: Just like the release of electrolytes into fluids

22. S1: But even, think about cholera, go back to epithelium.

23. S4: Yeah, with the zona pellucida

24. S3: with the junctions

25. S1: Yeah, when it breaks the junction, and by breaking the cells the water comes in, so these two are related.

Shortly after TS11, an hour and seven minutes into this session, the teacher asks how much of the lecture has been covered. The group members reach consensus that they are only a quarter way through this three-hour lecture topic and feel that there is still a lot left to cover. At this point, the teacher presses them to be explicit about what is missing from their concept map. This final sample, TS12, occurs 14 minutes later.

Talk Sample 12.) *T5, concept mapping reflection, 6th group meeting*

1. T5: Ok. Now how far have we got in the lecture?

2. S5: I think we’re done.

3. T5: Ok. So when you said we were only a quarter done, about ten minutes ago, we must have been considerably more than a quarter done.

4. S5: Or we worked really fast. [*ss laughter]*

5. T5: Or we worked really fast. So which was it?

6. S5: A little bit of both, I think.

7. T5: Well, how is it that we worked faster?

8. S3: Cos a lot of the things

9. S6: were organized.

10. S4: We had a certain ending

11. T5: We had something going. It takes times to get started, and I think that’s okay. Okay, so now an obvious next question is what happened in this session?

12. S1: We found out that we need to be organized, I found out, I’ll speak for myself.

13. S5: There’s weak areas, where we had a hard time

14. S3: And also doing it like this make me see the big picture more clearly.

15. T5: Are we missing details?

16. S3: Yeah.

17. S5: This is kind of like an overview, you’re still going have to study on your own.

18. S1: But not the itty bitty details though, cos we covered pretty much, like G_i_, G_s_,

19. S5: That’s true. Um, what else?

20. S1: The cyclic AMP can have an effect on the smooth muscle, can it?

21. S3: mhm.

22. S4: It’s up there, we got that already.

23. S1: Exactly, so … that’s not bad. And it’s still part of that, like a big picture, it’s already there.

24. S5: Yeah. I guess. Was it beneficial? Do you guys understand it better now?

25. S3: I could

26. S6: I think so. It’s clearer.

27. S4: Yeah, I think so.

28. S6: I think the best is to study with someone, cos there’s maybe a lot of things I thought I knew here, and it’s like, whoa…

29. S4: I think one good thing about this that I noticed, is that leaving out the details forces you to remember, and to recall, and I never do that, I always write down all the little details.

30. T5: But you can trust yourself.

31. S4: Yeah

32. T5: Cos actually it’s quite interesting how many details you do know. I think, you know a lot of details, if you can use triggers. And how much more detail do we need than this, I wonder, really? I bet, when you do the practice questions, you’ll discover one or two details that we did miss and should have been in, but not many.

The comment at TS12.U3 stems from contextual knowledge of at-risk medical students, and is used to elicit discussion on a common issue: the overestimation of how much they have to do often leads to paralysis, making it worth pressing them to objectively account for the volume of work confronting them, so that it can then be divided and managed. When S5 jokingly offers an alternative explanation (U4), the teacher embraces his implicit challenge and expects elaboration (U5, U7), leading into thoughtful, collaborative response (U8 – U10) that is aided by a further prompt to reflect on the whole session (U11). The question about missing details (U15) similarly displays strong knowledge of the learners: fear of missing details that might feature on exams, and inability to select what is important, is overwhelming for students, and results in copious unmanageable notes, often blindly copied from the lecture handouts. The sequence from U16 to U29 shows the group grappling with this difficulty, and by a process of social regulation they reach the explicit conclusion (U29) that excluding detail brings its own benefits. Note also how S5, who had been a prime doubter of this process, takes on the role of facilitator (U24), an example, perhaps, of goal contagion [72], whereby group members become infected with each other’s goals, so that the glow of successful task completion spills over into positive affect that imbues subsequent work. The last comment (U32) generalizes the students’ conclusion, and links back to a previously taught method of identifying and plugging knowledge gaps, a metacognitive linking moment that we have seen to be a hallmark of teaching expertise in this context.

Further examples could undoubtedly reveal additional insights into remediation of at-risk medical students, but these dozen talk samples hopefully provide adequate flavour of the patterns of interaction in these classrooms. We finish this section with one brief, final example:

Talk Sample 13.) *T5, at the beginning of 21st session*

1. T5: Okay, so any questions or comments or problems or anything? *[4 sec pause]*

2. S3: Oh, I have a comment. I was just finding it interesting yesterday some of my friends from second semester were telling me things about the GI and renal things and stuff, and I just kept asking them so why does it do that or why is it like that and they were like we don’t know, it just does, and it was just funny because I feel like, now I’m curious about it and I’m asking questions.

## Discussion

Before offering a summary of the key points raised in the results section, and discussing how they relate to our original aims and developing theory, we sound a brief note of caution. Having drawn on education theory to create a remediation programme [5], and then extended remediation theory based on participant perspectives and descriptions of practice [6,7], we discovered that although participants offered similar views of their experience, their outcomes differed considerably [7]. In this paper we have sought to disambiguate the language of that theory by sharing exemplars of classroom talk. In attempting to summarise our findings, we are in danger of re-creating that ambiguity by replacing the source of our understanding with descriptive labels and generalization [73,74]. But the function of talk is embedded in its context [52,57], language and knowledge are inseparable from the interactions in which they emerge [75,76]. Abstracting to theory from practice always risks producing a false dichotomy, as the two can only be fully understood in conjunction, through experience [25,36]. The following needs to be understood in light of this caveat, as a linear presentation, due to the constraints of language, of fundamentally interconnected ideas.

The clear differences in the practice of experienced and inexperienced teachers that have emerged from this analysis are summarized in Table 2; discussion of these will lead us towards our primary aim of revisiting our theory of remediation with new appreciation. While a direct causal link cannot be proven, the conclusion that differences in classroom behaviours contribute to differences in student outcomes seems intuitively, and theoretically, probable. All the teachers made efforts to validate the challenges these students face, to promote attribution of these difficulties to controllable behaviours [77], to encourage higher self-expectations [78], and to nurture the trust and positive attitude necessary for successful learning [79,80]. However, the less experienced teachers typically tended to overvalue compassion and affect [81,82], to trust too much, to inadvertently endorse unproductive framing of learning as the classroom game of passing the next exam [67]. Given that the short-term outcomes for both groups of teachers are similar [7], this risks potentially setting up many of these students for future difficulty, a failing of many remediation efforts [4]. In contrast, we saw the more experienced teachers foster genuine curiosity and will to learn [44,75,83], in part by refocusing discussion towards appreciative enquiry of specific behaviours and encouraging displays of intellectual enthusiasm, an approach more likely to have long term effect.

**Table 2:** Summary of key differences between experienced and inexperienced teachers

***Trait/behaviour***	***Experienced teachers***	***Inexperienced teachers***
Nurturing mentor	High expectations; model and expect appreciative inquiry, curiosity and intellectual enthusiasm	Over-value affect; allow unproductive framing
Challenging, disruptive facilitation	Problematize; challenge claims; flag disagreements and inconsistencies; promote cognitive conflict	Sometimes probe and ask questions; trust student understanding; allow premature closure of discussion
Diagnostician	Note inaccurate language use; query student uncertainty; expect answers to questions	overlook critical details; under -appreciate importance of language
Management of group dynamic	Dialogic stance; demand student-student interaction; draw-in quieter voices	IRF typical, especially for process discussion
Metacognitive voice	Frequent metacognitive time-outs; explicit generalization of process; links course elements; links curriculum elements	Cursory attention, little probing into depth of process use
Pedagogical context knowledge	Practical wisdom; timing, format and content of interventions; knowledge of learners, institutional context and classrooms	Less adaptive, less able to deliver course flexibly, less improvisation

Another difference arose in the way the teachers challenged their students. When left to themselves, students tend to avoid cognitive conflict [84,85], display faulty logic, produce inconsistent arguments, and, as newcomers to medical discourse, frequently misuse language [24,44,86]. The inexperienced teachers often did not notice or challenge unjustified claims, unacknowledged disagreements, and inaccurate language use, and, when they did, failed to insist on immediate pursuit of the problem, resulting in unhelpful premature closure of discussion [87]. Requiring students to elaborate and articulate meaning is valuable for reducing misunderstanding and eliciting new insights [44,88,89], while probing and problematizing to create cognitive conflict are core to development of critical thinking and learning [25,27,35]. The experienced teachers consistently posed disruptive, ‘noisy’ questions [90,91], flagging student uncertainty, inconsistency and disagreement for further exploration. Notably, the format of the intervention, whether it was a closed or open question, simple or complex, subtle or overtly directive, a statement, or even non-verbal, was less important than the timing and context [57,92], the intent of the challenge and the tenacious expectation of a considered student response. These are features of a dialogic stance on teaching [35,57] in which careful listening, attention to detail [93,94] and the ability to follow the logic of, and diagnose errors in, students’ argument all contribute to the creation of a knowledge-building conversation. Whether or not these are skills that grow with a teacher’s experience or are coincidentally due to individual characters, they certainly appeared as differences in the classrooms of the teachers in this study.

Of course, these ways of constructing knowledge create and are created by interaction and effective dialogue [63,95], and we saw this as variation in the dynamics of different groups. A key advantage of group work is the opportunity for students to be confronted with multiple viewpoints, to think together and scaffold each other’s learning [89], especially important for students in academic difficulty [6,96]. The initiation-response-feedback (IRF) dialogue format that typified the less experienced teachers’ classrooms tends to locate authority in the teacher and reduces student-student interaction [69,97]. In contrast, the experienced teachers insisted that students comment on each other’s ideas and take ownership of the conversation, creating a discourse community of shared regulation [91,98]. Given the challenges of self-assessment [99], this promotion of social regulation offers students the chance to learn how to self-regulate through critiquing others [68]. It seems likely that fostering dynamic group interaction not only enables immediate collaborative learning, but also supports more independent self-regulation for improved longer-term outcomes.

The experienced teachers also put much more emphasis on ‘metacognition’, regularly inviting the students to examine and summarize their learning processes and classroom events, while frequently linking elements of the course and offering generalizations of techniques. The lack of this from inexperienced teachers misses a key objective of the course: raising awareness of the value of these skills [100] through explicit abstraction to new domains [88] is vital in persuading these students of the relevance of changing their behaviours, a necessary prerequisite for the long-term development of self-regulatory dispositions [101]. The way the experienced facilitators take on the role of the metacognitive, self-regulatory voice of their groups serves as a model for the students and appears to generate genuine shifts in attitudes and behaviours.

It seems that contextual expertise may tie together all the above techniques of experienced teachers. Knowledge of this course and the wider curricular and institutional context allows them to uncover links between disciplines, processes and concepts; knowledge of these students, and their particular struggles, enables them to induce students to make and appreciate these links, and to engage them in seeking behavioural change. For the practice of remediation, these are aspects of what we might call ‘*pedagogical context knowledge*’ [102]: understanding of learners, their experience, misconceptions and language use [18,21,44,75]; understanding of content [103], which for remediation includes the processes of learning, both cognitive and affective, as well as the content of the remediation course and its links to the whole curriculum; and understanding of the classroom [102], as action unfolds, complexly, moment by moment. Thoughtful application of this contextual knowledge is essential to the provision of effective support and requires teaching presence and practical wisdom [7]. Presence includes mindful awareness of and engagement with all the elements of classroom life [13], reflecting in action [104] to respond adaptively and flexibly to student needs as they emerge through interactions [102,105,106]. This ability to improvise [107] requires the practical wisdom to know when and how to act [18,39], a finely tuned contextual expertise necessarily borne of experience [11,75,108].

Thus, we feel that examination of these examples of classroom talk, and the differences between experienced and inexperienced teachers, has enabled us to address our initial aims, and offer a description of the theory/practice of successful remediation of at-risk medical students. Remediation, as with any form of education, is situated within complex pedagogic settings where details matter and small perturbations can have significant effects [27,109]. Since most learning is through language, the ‘tool of tools’ [25], small, stable groups can provide an ideal climate for students to support each other, for dialogue that develops self-regulatory skills through social regulation. This can happen when experienced teachers combine artistry [110], presence, practical wisdom and a dialogic stance [57] to create space for their group to become a community of inquiry [91] that expects high level interaction and critical thinking.

### Limitations and further work

There are, of course, a number of limitations with this work. To start with, education is inherently uncertain, and all descriptions can only be incomplete [60,111]. Furthermore, since small groups are complex dynamic systems, always changing because they are dependent on interactions [25,27], our recordings could only be transient moments in their unfolding evolution; further reducing those recordings to a handful of snapshots for this paper, through selection by one participant, inevitably weakens ability to generalize from these findings. Additionally, we have examined the work of only six teachers, all in the same institutional context; further work would need to explore similar work in other contexts. Although the correlation between experience and outcomes is strong, experience alone is unlikely to be enough: the experienced teachers in this study all chose to work in this context, and it is likely that desire and particular pedagogical beliefs are also required for successful remedial teaching.

Another limitation, that has the potential for interesting development, is the use of only audio recording. The use of video recording, not only for further research, but also as a tool for faculty development, has the potential to provide direct feedback to less experienced teachers drawn from their own classrooms [112,113]*.* One cannot know what one has failed to notice, unless it is pointed out. Classroom video recordings, used in collaborations between experienced and inexperienced teachers, could help inexperienced teachers learn to respond more adaptively to emerging classroom interactions and thus provide tangible long-term benefits to many struggling students. Such work may also enable further exploration of the mechanisms of social regulation, into how it might be developed and how it might result in improved self-regulation [68,114].

## Conclusion

Undoubtedly, reflection on personal experience of remediation teaching is the best way to understand it [25]. Lacking this, careful inspection and analysis of classroom discourse, which is an essential vehicle for teaching and learning in any context, can provide insight into the kinds of behaviours that embody effective remediation.

Such work is effectively carried out in a small, stable community of inquiry that requires participation of all group members in the generation of dialogue for collaborative knowledge construction and social regulation. Such a learning climate can evolve in groups led by experienced teachers with extensive pedagogical context knowledge and expertise. When these teachers pay attention to details of both content and process, they can use timely interventions to foster curiosity and the will to learn. These teachers should actively challenge students’ language use, logical inconsistencies and uncertainties, problematize their assumptions, and provide a metacognitive regulatory voice that can generate attitudinal shifts and nurture the development of independent critical thinkers.

This is no small task, but, if the large differences in outcomes are found in other contexts, then, in order to maximise the effectiveness of limited resources, it must be worth putting experienced teachers to work with the students who need them most.

## Abbreviations

TS: Talk sample; U: Utterance (talk turn); S: Student; T: Teacher; MCQ: Multiple choice question; IRF: Initiation-response-feedback; ELLS: Essential lifelong learning skills; MCAT: Medical college admissions test.

## Competing interests

The authors declare that they have no competing interests.

## Author notes

The research was conducted at Ross University School of Medicine, Dominica. At the time of data collection, KW was assistant professor in the Centre for Teaching and Learning at RUSM, and was director of the Essential Lifelong Learning Skills course, mandatory for students who failed and repeated a semester at the school.

## Authors’ contributions

KW performed this research as part of his PhD. KW recorded, transcribed and analysed the classroom talk, and is the principal author of this paper. AS and CVDV contributed important suggestions and comments at both the design-stage of the research and the editing stage of writing this paper. All authors read and approved the final manuscript.

## Pre-publication history

The pre-publication history for this paper can be accessed here:

http://www.biomedcentral.com/1472-6920/13/132/prepub
